# The efficacy and safety of electroacupuncture against urinary incontinence after stroke: A protocol for systematic review and meta analysis

**DOI:** 10.1097/MD.0000000000022275

**Published:** 2020-09-18

**Authors:** Peng Wang, Jiyuan Shi, Liang Zhao, Mengmeng Li, Jiawei Jiao, LingYun Li, Jinhui Tian, Shiguang Wang, Shanfeng Zhang

**Affiliations:** a College of Medicine, Zhengzhou University of Industrial Technology; b Basic Medicine Department, School of Nursing and Health, Zhengzhou University, Zhengzhou; c Evidence-Based Nursing Centre, School of Nursing; d School of Basic Medical Sciences, Lanzhou University, Lanzhou City, Gansu Province; e Experimental Center for Basic Medicine; f Biochemistry and Molecular Biology, Zhengzhou University, Zhengzhou, Henan, China.

**Keywords:** electroacupuncture, meta-analysis, stroke, urinary incontinence

## Abstract

**Background:**

Urinary incontinence (UI) is still a persistent challenge in many stroke survivors, affecting the quality of life and emotional being of these individuals. Numerous studies have demonstrated the curative effect of electroacupuncture on post-stroke incontinence, however they were mired with questionable quality and inconsistencies in safety and efficacy. Therefore, the main objective of this meta-analysis is to provide a comprehensive evaluation of the efficacy and safety of electroacupuncture against urinary incontinence after stroke, with a view of providing more reliable evidence-based solutions for UI.

**Methods:**

A systematic literature search will be conducted using PubMed, EMBASE, the Cochrane Central Register of Controlled Trials, Web of Science, and 4 Chinese databases from inception to June 2020 to identify randomized control trials that report on electroacupuncture against urinary incontinence after stroke. Two reviewers will independently identify eligible studies and extract data. The risk of bias of the included randomized control trials will be evaluated according to the Cochrane tool. Risk ratio and 95% confidence intervals will be used to estimate the efficacy of treatment,. and the grading of recommendations, assessment, development, and evaluation approach to rate the certainty of evidence. The statistical heterogeneity will be evaluated by Cochran's Q and the I^2^. Data will be analyzed using Stata software (Version 13.0, Stata Corp, College Station, TX, USA).

**Results:**

This study will provide a comprehensive evaluation of the efficacy and safety of electroacupuncture against UI after stroke, with a view of providing more reliable evidence-based solutions for UI.

**Ethics and dissemination:**

This work synthesises evidence from previously published studies and does not require ethics review or approval. A manuscript describing the findings will be submitted for publication in a peer-reviewed scientific journal.

**INPLASY registration number:**

INPLASY202050073

## 1 Introduction

According to the International Continence Society, urinary incontinence (UI) is as any involuntary leakage of urine. Urinary incontinence after stroke is a common complication that develops in patients with stroke.^[1]^ Indeed, globally statistics show that 28% to 79% of stroke survivors develops urinary incontinence.^[2]^ Stroke damages neurological lesions in the brain, which impairs detrusor function in the survivors. For post stroke patients (PUR), UI remains one of the most common complications associated with high morbidity, disability, and institutionalization rates.^[2,3]^ UI is still a persistent challenge in many stroke survivors, affecting the quality of life and emotional being of these individuals.^[3,4]^ UI is also associated with several psychological problems such as anxiety and depression.^[5]^ Interestingly, the development of UI following an acute stroke is also a marker for prognosis of the complication.^[4,5,6]^ Here, UI is strongly associated with both high mortality rates and poor functional recovery. Accordingly, the management or treatment of post-stroke UI continues to attract a lot of clinical interest.^[4]^

Management of post-stroke detrusor overactivity is therefore an important aspect in rehabilitation care.^[4]^ Recommended treatments for post-stroke UI include behavioral techniques (bladder training, timed or prompted voiding, fluid and diet management and environmental, and lifestyle support), exercises for pelvic floor muscles, electrical stimulation, medications such as anticholinergics and alpha-blockers and use of medical devices (indwelling urinary catheter or external collecting device, interventional therapies, surgery, absorbent pads, and catheters), however, none of these interventions provides sufficient remedy.^[4–9]^ Most of them are inefficient with several limitations, thus optimal solutions for post-stroke urinary incontinence remains elusive.^[4]^

Acupuncture is a typical traditional Chinese medicine treatment for migraines, widely applied in clinical setting,^[10]^ and it is recommended as a supplementary or alternative treatment for various diseases.^[11–14]^ EA is a form of acupuncture, consisting of electrical impulses passing through needles to stimulate acupoints.^[4]^ Although numerous studies have demonstrated the curative effect of EA on post-stroke incontinence, they were mired with questionable quality and inconsistencies in safety^[15]^ and efficacy.^[4]^

Therefore, the main objective of this meta-analysis is to provide a comprehensive evaluation of the efficacy and safety of electroacupuncture against UI after stroke, with a view of providing more reliable evidence-based solutions for UI.

## 2 Methods/design

### 2.1 Design and registration

This study shall embrace the reporting Items for Systematic Reviews and Meta-Analyses (PRISMA) for systematic reviews (SR) and meta-analysis (www.prisma-statement.org). The systematic review and meta-analysis will be performed and reported based on PRISMA guidelines as well.^[16]^ This SR is registered on International Platform of Registered Systematic Review and Meta-analysis Protocols (INPLASY.COM). The registration number is INPLASY202050073.

## 3 Eligibility criteria

### 3.1 Types of studies

Studies on randomized control trials (RCTs) on optimal EA treatment for PUR will be included. Although the selection of the studies will be limited to clinical subjects, there will be no restrictions with regard on language, or publication date. Non-randomized trials, clinical studies, quasi-RCTs, cluster RCTs, observational studies, animal experimental models, qualitative studies, letters to the editor, news articles, review articles, editorials, case series, case reports, commentaries studies, and duplicate publications will all be excluded.

### 3.2 Types of participants

Patients included in post-stroke clinical trials (both experimental and control groups) must have been diagnosed with PUR based on standardized diagnostic criteria. The diagnosis must have been validated with cerebral hemorrhage or cerebral infarction using CT or MRI. There are no limitations on age, gender, education status, or ethnic background. However, participants must be mentally sound without cognitive impairment, mental disorder, and other severe underlying comorbidities. Studies on patients with serious systemic or neurologic disease, urinary system infection, undergoing preoperative radiotherapy, or chemotherapy, a combination of serious risks such as cardiovascular, liver, kidney, and hematopoietic system, or refused to accept acupuncture treatment will also be excluded because of confounding potential of these conditions.

### 3.3 Types of interventions

EA should be the therapeutic intervention applied in the experimental group. Individuals who underwent EA in combination with other therapies will only be included if the combined therapy have the both same groups. However, other acupuncture methods (nonelectroacupuncture) and dry needling not based on oriental medicine and meridian theory will be excluded. No specific criteria regarding the needle size, acupoint selection, current stimulation frequency, intensity, retention time, and treatment course is set. All manipulations such as administration of placebos, psychological control, drug therapy, or physical therapy modalities will be performed by a general physician. Studies in which the control group received recommended care will also be included in this study.

### 3.4 Types of outcome measures

The primary indicators for the efficacy of EA therapy will be the volume of residual urine, time to first urination, bladder capacity, urinary flow rate, and urine output.

Secondary indicators will include adverse events such as (Death, infection, acupuncture sickness, severe pain etc).

## 4 Search strategy

Relevant studies published by June 2020 will be independently and systematically searched in appropriate databases by 2 researchers. The search shall be made in 4 international, and 4 Chinese databases. The selected databases include PubMed, EMBASE, the Cochrane Central Register of Controlled Trials, Web of Science, Chinese databases China National Knowledge Infrastructure, Chinese Scientific Journal Database, SinoMed, and Wanfang. Relevant studies must have assessed the safety and efficacy of EA on PUR. The key search words are electroacupuncture, urinary retention, retention urinary, postoperative urinary retention, and stroke. The full search strategy for PubMed is provided in Table 1, and similar strategies will be applied to the other electronic databases. References in related systematic reviews and clinical guidelines will also be searched to identify potential literature relevant to this study.

**Table 1:**
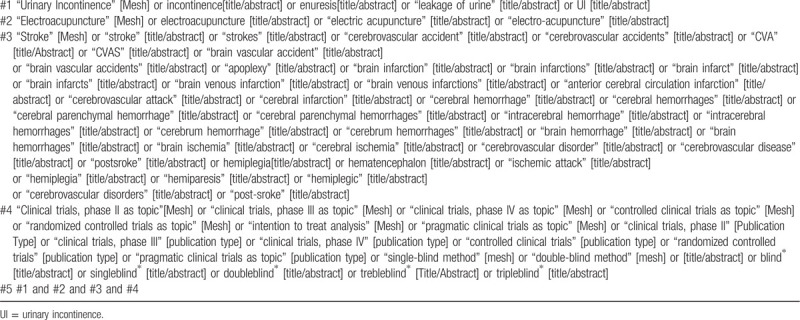
Search strategy of PubMed database.

### 4.1 Study selection and data extraction

The data will be organized using the ENDNOTE X7 referencing software (Endnote version 7, Thomas Reuters, USA) literature management software. Studies meeting our inclusion criteria will be retrieved independently by 2 investigators who shall systematically screen through titles, abstracts, and keywords of the searched articles. The titles and abstracts for articles not meeting the eligibility criteria will be excluded. Relevant articles will be reviewed to extract significant findings. The extracted data will be organized and compiled by the 2 investigators, independently. Any disagreement regarding inclusion of some studies will be resolved by discussion and consensus between the 2 reviewers. If this failed, it shall be resolved by an arbitrator.

Extraction of data from the original articles will be guided by a predetermined data extraction form. Information to be captured in the form include name of the first author, study location (country), year of publication, the total number of participants, study design, sample sizes, diagnostic criteria, the details on intervention, and control group, measurement for the main success indicators for positive outcome and cases of adverse events. If the data in a study are insufficient or ambiguous, the corresponding author will be contacted to provide additional information.

### 4.2 Assessment of risk of bias in the included studies

Two independent reviewers will assess the risk for bias based on the Cochrane Handbook version 5.1.0,^[17]^ which evaluates selection, performance, detection, attrition, reporting, and other bias.^[18]^ The methodological quality will be classified in 1 of the 3 categories; high, unclear, or low risk. Any discrepancies will be resolved by a third reviewer.

We classify each study as low-bias risk according to the following criteria:

(1) all 6 items are low-bias risk,
(2) 1 or 2 of the 6 items are assessed as ambiguous risks, and the other items are low Risk of bias. If the study does not meet the low bias criteria, we believe that the study has a high risk of bias.^[19]^

## 5 Statistical analysis

Statistical analysis will be performed using the Stata software (Version 13.0, Stata Corp, College Station, TX, USA). Risk ratio and 95% confidence intervals will be used to estimate the efficacy of treatment, and result for the meta-analysis will be presented using the forest plot. Cochran's Q and the I^2^ statistic will be used to evaluate the statistical heterogeneity. When the *P* < .05 and I^2^ > 50%, the heterogeneity will be considered significant. Random-effects model will therefore be used for the pooled estimates and subgroup analyses. Conversely, if *P* < .05, and I^2^ < 50%, then the heterogeneity shall be considered not significant, thus the fixed effects model shall be used in the subsequent meta-analysis. Two reviewer will independently use the grading of recommendations, assessment, development, and evaluation approach to rate the quality of evidence as high, moderate, low, or very low.^[20,21]^

The publication bias or heterogeneity will be evaluated using the symmetry of the funnel plot and Egger test. In Egger test, bias will be significant when *P*-value < .05.

### 5.1 Subgroup analysis and sensitivity analysis

If substantial heterogeneity is found, subgroup analysis will be performed along the gender, age, acupuncture types, and different electroacupuncture acupoints. Moreover, if the number of included studies will be more than 10 but a substantial heterogeneity is observed, then meta-regression analysis will be performed to explore the potential source of the heterogeneity. In the sensitivity analysis, studies will be excluded one at a time. The remaining studies be then analyzed to estimate the impact of the omitted research on the overall results.

### 5.2 Patient and public involvement

No patients or public are involved.

## 6 Conclusion

Urinary incontinence is a common complication after stroke. It seriously hinders the rehabilitation process of patients with stroke, affects their quality of life and mental health and imposes a tasking burden to the nursing staff. Currently, although multiple studies have reported on the role of electroacupuncture on the effectiveness of therapeutic interventions during post-stroke urinary incontinence, there is no systematic review on the effectiveness and safety of electrotherapy for post-stroke urinary incontinence.

The work we propose has several potential limitations. First, the findings might be influenced by inherent deficiencies in the existing published literature, because the measurement of treatment outcomes after electroacupuncture in post-stroke urinary incontinence varies considerably across studies. Second, because this study will only include RCT publications for reliability reason, there is a possibility that the study sample will not be representative of the overall population. Finally, if the number of studies to be included shall be small, efforts to explore heterogeneity and performing a meta regression analysis shall be limited. Meanwhile, if the degree of clinical heterogeneity shall be significant, then the reliability of the results will be questionable.

## Acknowledgments

The authors are thankful to Dr Jinhui Tian who provided valuable feedback on the draft version of the protocol.

## Author contributions

JS, JT, and PW conceived the idea for this study; JS, LZ and SZ designed this protocol. JS and LZ drafted the protocol. SW and JT reviewed the protocol and provided critical feedback. All authors approved the article in its final form.

## Abbreviations

Abbreviations: PUR = post stroke patients, RCTs = randomized control trials, UI = urinary incontinence, UIAS = urinary incontinence after stroke.
